# La gestion des données du système d’information sanitaire de routine en période de crise sécuritaire dans le district sanitaire de Tombouctou, au Mali en 2023

**DOI:** 10.11604/pamj.2024.47.180.42772

**Published:** 2024-04-11

**Authors:** Bocar Mahamane Traore, Aminata Traore, Amadou Tila Kebe, Kizito Dabou, Djibril Kassogue

**Affiliations:** 1 Département du Système Local d'Information Sanitaire, Centre de Santé de Reference, Tombouctou, Mali,; 2 Momentum Integrated Health Resilience, Bamako, Mali,; 3 Département Santé, Direction Régionale de la Santé, Tombouctou, Mali,; 4 Département Santé, Direction Régionale de la Santé, Tombouctou, Mali,; 5 Département Santé, Hôpital de Tombouctou, Tombouctou, Mali

**Keywords:** Crise sécuritaire, déterminants, gestion des données, système d’information sanitaire de routine, Tombouctou, Mali, Security crisis, determinants, data management, routine health information system, Timbuktu, Mali

## Abstract

**Introduction:**

un système d'information sanitaire (SIS) efficace garantit la production, l'analyse, la diffusion et l'utilisation d'informations fiables et actualisées sur les déterminants de la santé. Cependant, il peut rencontrer des obstacles qui entravent son fonctionnement, il en est ainsi des conflits armés, qui limitent l'accès et la qualité des services des soins. Notre étude visait à contribuer à l'amélioration de la gestion des données du système d'information sanitaire de routine dans le district sanitaire de Tombouctou en situation de crise sécuritaire.

**Méthodes:**

une étude transversale descriptive, réalisée du 15 avril au 08 septembre 2023 dans le district sanitaire de Tombouctou auprès des agents impliqués dans le SIS. Les données obtenues à partir d'un questionnaire d'enquête ont été analysées sur Epi Info version 7.2.2., et traitées sur Microsoft Word et Excel 2016.

**Résultats:**

au total, 6 formations sanitaires ont été enquêtés. Les niveaux de collecte, d'analyse et de feedback étaient à des taux très faibles. Par rapport à la qualité des données: 100% de complétude, 92,40% de promptitude et 68,11% d'exactitude. Les contraintes majeures étaient: faible implication des agents de santé dans le SIS (22,22%), insuffisance de formation sur le Système d'Information Sanitaire de Routine (SISR) (29,63%), la supervision (47,06%), l'inaccessibilité à internet (66,67%), le sentiment d'insécurité (37,04%) et la peur (61,76%) dans les formations sanitaires.

**Conclusion:**

nos résultats présentent un faible niveau de processus, une mauvaise couverture réseau, une insuffisance en personnel qualifié pour la gestion du SIS et une insécurité grandissante. Une étude mixte plus large permettrait de mieux les comprendre.

## Abstract (en)

**Introduction:**

an effective health information system (HIS) ensures the production, analysis, dissemination and use of reliable and up-to-date information on the determinants of health. However, it can encounter obstacles that hinder its functioning, such as armed conflicts, which limit access and quality of healthcare services. The purpose of our study was to help improve data management for routine health information system in the health district of Timbuktu during a security crisis.

**Methods:**

we conducted a descriptive cross-sectional study, among health information management professionals in the Timbuktu Health District from 15 April to 08 September 2023. Data obtained from a survey questionnaire were analyzed using Epi Info version 7.2.2. and processed using Microsoft Word and Excel 2016.

**Results:**

a total of 6 health facilities were surveyed. Data collection, analysis and feedback were very poor. Data quality was 100% complete, 92.40% prompt and 68.11% accurate. The major constraints were: low involvement of health workers in the SIS (22.22%), insufficient training on the SISR (29.63%), supervision (47.06%), internet inaccessibility (66.67%), feeling of insecurity (37.04%) and fear (61.76%) in health facilities.

**Conclusion:**

our results show low-level processes, poor network coverage, shortage of qualified health information management professionals and increasing insecurity. A broader mixed-methods research would provide a better understanding.

## Introduction

Un système d'information sanitaire (SIS) efficace garantit la production, l'analyse, la diffusion et l'utilisation d'informations fiables et actualisées sur les déterminants de la santé [1]. L'Organisation mondiale de la Santé (OMS) considère le SIS comme un des six piliers essentiels du système de santé pour atteindre l'objectif de «la santé pour tous» [2]. Des données de haute qualité soutenant les décisions de gestion de la santé sont essentielles à une gouvernance, un leadership et une gestion efficace [3,4].

Les conflits armés ont des effets négatifs importants sur les systèmes de santé, limitant l'accès et la qualité des services préventifs et curatifs [5]. En plus, ils surviennent de manière disproportionnée dans des pays où les systèmes d'information sur la santé publique sont déjà faibles [6]. Les conflits armés en cours au Soudan du Sud, en République centrafricaine, dans le nord-est du Nigéria et dans d'autres pays africains ont par ailleurs eu des conséquences similaires [7].

Les systèmes d'information se sont fortement développés au cours des vingt dernières années dans le système de santé des pays développés [8] tandis que ceux des pays en développement sont faibles, fragmentés avec des données collectées de manière incohérente et désorganisée; que leurs sources d'informations premières sont parfois dispersées, isolées et difficiles d'accès et qu'ils sont en sous-effectif et dotés de ressources insuffisantes [9]. Dans de tels pays, des obstacles au développement du SIS peuvent exister à chaque étape du processus (collecte de données, analyse, rapport, etc.), exacerbés par le manque de ressources matérielles (outils informatiques), des ressources humaines insuffisantes et des exigences de rapports incohérentes [10]. Les agents de santé reçoivent rarement de rétro-informations sur le SIS, et lorsqu'elles ont été fournies, elles ont eu tendance à être négatives, obsolètes et improductives [11].

Si l'on consulte la base bibliographique «PubMed» à la rubrique «routine health information system» on dénombre, en juin 2023, 8073 publications dont 5354 entre les années 2013 et 2023, contre 2719 entre 1970 et 2012. Si on change la rubrique en «Crisis Security» en ce qui concerne le contexte, on dénombre 2150 dont 1593 entre 2013 et 2023 et seulement 557 entre 1950 à 2012. Ces chiffres dénotent aussi de l'intérêt des scientifiques pour faire avancer les connaissances dans le domaine. Pour renforcer le SIS, l'OMS et ses partenaires ont lancé le cadre du « Réseau de métrologie sanitaire » en 2005 dont l'objectif est d'aider les pays et les partenaires à produire et à utiliser des données de meilleure qualité pour prendre des décisions fondées sur des bases factuelles [12]. Le cadre Performance of Routine Information System Management (PRISM) a été développé sur la base des faiblesses documentées du SISR, une approche innovante pour concevoir, renforcer et évaluer le SISR [13].

Depuis 2007, certains pays africains ont pu utiliser ces deux outils pour évaluer leurs systèmes d'informations sanitaires. Ce sont notamment le cas du Burkina Faso, Burundi, Côte d'Ivoire, Madagascar, Gabon, République Démographique du Congo (RDC), Mali, etc. [14-19]. Ces dernières ont permis de mettre en évidence un certain nombre de faiblesses: une faible promptitude et complétude des rapports, des incohérences des données, la faible sécurisation des données, la faible analyse des données [20].

Le Mali connaît, depuis 2012, une crise sociopolitique et sécuritaire majeure en particulier les régions du nord dont le district sanitaire de Tombouctou [21]. Cette crise a entraîné la suspension de l'aide au développement accordée par certains partenaires techniques et financiers. Ceci a influencé négativement le financement des activités de la santé [21]. L'accès aux soins des populations affectées par la crise a été restreint par les destructions et/ou pillages des infrastructures de santé (18,6%) [21]. Le Mali dispose depuis 1998 d'un Schéma Directeur du Système National d'Information Sanitaire et Sociale (SD-SNISS) qui a fait l'objet de plusieurs évaluations en 2003, en 2009, en 2013 et la dernière en 2018 avec l'outil PRISM [22-24]. En août 2015, le Mali a adopté le logiciel Dhis2 pour la gestion de ses données sanitaires de routine appuyé jusqu'en mars 2020 par MEASURE Evaluation [25,26]. Son utilisation a permis au Ministère de la Santé d'améliorer la collecte, la transmission, la revue, l'analyse, la sécurité, la disponibilité, la confidentialité et la qualité des données nationales [25].

La gestion des données SISR et ses déterminants en situation de crise sécuritaire en Afrique sont peu connus. En effet, la plupart des recherches dans ce domaine se sont intéressées à l'impact des conflits sur les prestations de programmes de santé. La présente étude vise à donner un aperçu des facteurs qui influencent le fonctionnement optimal du SIS dans le contexte d'insécurité. Pour toutes ces raisons, nous avons réalisé en 2023 dans les formations sanitaires du district sanitaire de Tombouctou auprès des personnes chargées du SIS une étude sur la gestion du SISR en période de crise sécuritaire avec pour objectifs de décrire le fonctionnement du SIS en temps de crise sécuritaire, d'analyser la qualité des données en termes de taux de complétude, de promptitude et d'exactitude dans le district sanitaire de Tombouctou et d'identifier les contraintes majeures et les stratégies de résilience des acteurs du SIS pour maintenir la performance du SIS en situation de crise sécuritaire.

En atteignant cet objectif, nous répondons aux questions de recherche suivantes: 1) Comment le SISR fonctionne-t-il en temps de crise sécuritaire ? 2) Quels sont les taux de complétude, de promptitude et d'exactitude ? 3) Quelles sont les contraintes majeures et les stratégies de résilience des acteurs du SIS pour maintenir la performance du SIS en situation de crise sécuritaire.

## Méthodes

**Type d'étude:** il s'agit d'une étude transversale descriptive dans les formations sanitaires du district sanitaire de Tombouctou auprès des personnes chargées du SIS.

**Site d'étude:** l'étude a été réalisée dans 6 formations sanitaires dont 3 en milieu urbain et 3 en milieu rural dans le district sanitaire de Tombouctou au nord du Mali. Le District sanitaire de Tombouctou couvre une superficie de 347 438 km^2^ soit 68,8% de la superficie de la région et 28,02% de la superficie du pays avec une densité de moins d'un habitant /km^2^ et une population de 189 096 habitants (source RGPH 2009 appliqué au taux d'accroissement). La collecte des données s'est déroulée du 23 mai au 14 juin 2023.

**Collecte des données:** la méthode utilisée était une enquête diagnostique à travers une interview face à face avec les agents impliqués dans la collecte des données du SIS. Le questionnaire pré établi à cet effet a été administré individuellement à tous les agents qui sont impliqués dans la gestion des données. Les enquêteurs (2) préalablement formés sur les outils ont appuyé l'enquêteur principal dans la collecte des données sur le terrain. Les tâches des enquêteurs se résumaient à la collecte et à la vérification des données, ils se sont assurés qu'elles soient complètes, précises et correctement enregistrées. Les données ont été collectées avec le logiciel Kobocollect installé sur des smartphones. Le questionnaire a été préalablement testé et validé dans une formation sanitaire non tirée au sort pour l'étude.

**Participants de l'étude:** notre population était constituée par les personnes chargées du SIS dans les formations sanitaires tirées au sort. Étaient inclus dans l'étude les formations sanitaires fonctionnelles tirées au sort et les agents de santé impliqués dans la collecte des données dans les formations sanitaires tirées. N'étaient pas inclus dans l'étude les formations sanitaires non-fonctionnelles, ainsi que celles fonctionnelles refusant de participer à l'étude.

**Échantillonnage:** il s'agit d'un échantillonnage aléatoire simple selon un choix raisonné. Les formations sanitaires du district sanitaire ont été réparties en milieu urbain et rural. Comme il n'y a que 3 formations sanitaires au niveau urbain, ces dernières ont été automatiquement sélectionnées. La méthode d'échantillonnage aléatoire simple a permis de sélectionner 3 formations sanitaires sur les 15 au niveau rural. Ainsi, les six formations sanitaires sélectionnées ont été enquêtées.

**Variables/données collectées:** les principales informations ont été collectées à l'aide d'un questionnaire élaboré à cet effet inspiré des outils révisés PRISM version 3.1. Pour cela, des observations, des entretiens structurés, des abstractions de données et des examens de documents guidés par une liste de contrôle d'observation ont été conduits pour recueillir les informations pertinentes auprès de tous les agents des formations sanitaires sélectionnées qui sont impliquées dans la gestion des données. Les informations collectées étaient les suivantes: a) les données sociodémographiques (âge, sexe, résidence, années d'emploi au SIS, diplôme professionnel des répondants et nombre de personnels impliqué dans la collecte des données du SISR); b) les données sur les aspects importants du fonctionnement (collecte, traitement, analyse, transmission, saisie dans le DHIS2, présentation et retro-information); c) les informations sur la qualité des données (taux de complétude, promptitude et exactitude ou vérification des données) et; d) les contraintes majeures liées au SISR (contraintes liées au personnel, à la formation, la supervision et la retro-information, à la connexion et à la sécurité). Pour la vérification (taux d'exactitude) des données, 3 mois ont été sélectionnés: octobre, novembre et décembre 2022.

### Définitions opérationnelles

***Taux de complétude des rapports (%):*** c'est le nombre des rapports qui sont complets (tous les éléments de données sont remplis) sur le nombre total des rapports disponibles ou reçus.

***Taux de promptitude des données (%):*** c'est le nombre des rapports envoyés ou reçus dans les délais sur le nombre total des rapports disponibles ou reçus. Pour notre étude, nous avons la date de saisie dans le DHIS2 qui avant le 15 du mois prochain pour chaque mois.

***L'exactitude ou vérification des données (%):*** il compare les données recomptées aux données rapportées. Ce dernier est dit exact ou normal s'il est compris entre 90% et 110%. On parle de bonne qualité, lorsque les données répondent aux critères des trois dimensions de qualité: exactitude entre 90% et 110%, complétude = 100% et promptitude ≥ 90%. On parle de mauvaise qualité, quand les données ne correspondent pas à ces critères: exactitude <90% ou >110%, ou complétude <100%, ou promptitude <90% [27-34].

**Sources des données:** sont la base de données Dhis2, les rapports mensuels d'activités (RMA) pour les périodes concernées et les registres de consultations des services ciblés (registres de consultations prénatales (CPN), nutrition, consultation curative et pointage de la vaccination des enfants pour les périodes retenues).

**Indicateurs de revue:** les indicateurs suivants ont permis de vérifier les taux de complétude, de promptitude et d'exactitude des données: malnutrition aiguë sévère sans complication, le nombre de femmes ayant au moins bénéficié de quatre CPN, le nombre de cas de paludisme simple chez les moins de 5 ans traités avec les combinaisons thérapeutiques à base d'artémisinine (CTA) et le nombre d'enfants de moins d'un an ayant bénéficié des doses de Penta 3.

**Gestion des biais:** les milieux urbain et rural ont été pris en compte pour avoir une similarité selon le milieu. Toutes les personnes chargées du SIS ont été concernées dans notre étude. Ces deux actions sont de natures à amoindrir les biais de sélection et d'information qui peuvent être associés aux sources de données et aux répondants à l'enquête. La qualité des données a été contrôlée à plusieurs niveaux pour assurer leur fiabilité. Pour certains Items, les enquêteurs se sont limités aux seules affirmations des répondants sans autres sources de vérification, c'est le cas notamment des contraintes sécuritaires. L'insécurité étant notre difficulté majeure, nous avons mis en place des mesures de mitigations pour assurer la sécurité de l'équipe mais aussi des répondants à notre enquête.

**Analyse des données:** les données ont été extraites sous forme de fichier Excel à partir du logiciel Kobocollect. Les données ont été ensuite exportées puis analysées par le logiciel Epi Info version 7.2.2. Les résultats ont été présentés sous forme de fréquences et de pourcentages. Les mesures de tendances centrales et de dispersions (Moyenne, Écart type) ont été utilisés pour la description des variables quantitatives. Le traitement de texte, la réalisation des tableaux et des graphiques ont été faits à l'aide du logiciel Microsoft Word et Excel 2016.

**Considérations éthiques et administratives éventuelles:** le protocole a été validé par les autorités compétentes de la région de Tombouctou. Le consentement éclairé a été demandé et obtenu de manière écrite de la part de tous les participants. La confidentialité des données a été garantie et les résultats ont été présentés lors d'un atelier d'un jour puis diffusés après validation au niveau du district sanitaire de l'étude.

## Résultats

**Caractéristiques sociodémographiques:** au total, 6 formations sanitaires (3 urbaines et 3 rurales) ont été enquêtées et 27 agents de santé impliqués dans la collecte des données du SISR ont répondu à notre questionnaire d'enquête. Sur l'ensemble des répondants, l'âge moyen était de 37±9 ans et la classe d'âge la plus représentée était celle des 25-34 ans (56%). Plus de la moitié (59%) des répondants était du sexe féminin avec un sex ratio H/F à 0,69 en faveur de la femme et 56% résidaient en milieu urbain. Plus du tiers (41%) des répondants étaient des infirmières avec une expérience professionnelle moyenne de 7±4 ans. La moyenne des répondants était de 5,4±2 par formation sanitaire dont celle de Hondoubomo Koïna était la plus représentée (26%). Plus du 1/4 (26%) des répondants avaient un poste de responsable de formation sanitaire (directeur technique du centre ou suppléants).

**Figure 1:**
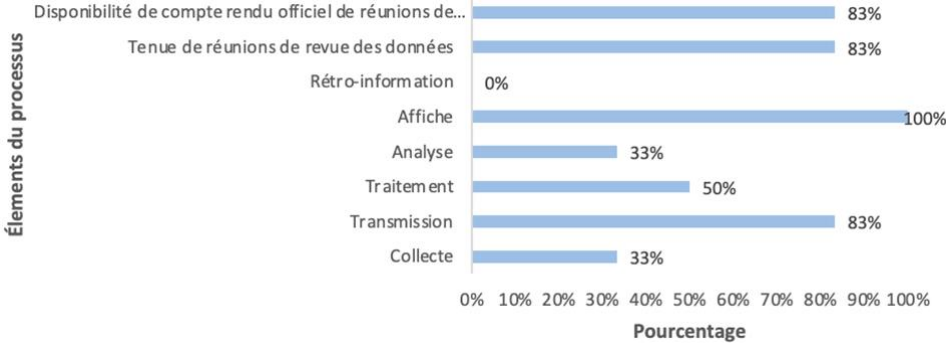
processus de fonctionnement du SISR

**Processus de fonctionnement du SISR:** l'étude avait noté une disponibilité des directives nationales à 83%, des supports en stock suffisant à 100%, des copies des rapports à 100%, ainsi qu'un taux de rupture des supports à 17% sur les six derniers mois. Les directives nationales en matière de collecte, d'analyse et de rétro-information sont à des niveaux très faibles (moins de 50%) pour des scores relativement meilleurs pour le traitement, la transmission, l'affichage des données, la tenue et la disponibilité des comptes rendus des réunions (supérieur ou égal à 50%) (Figure 1). Le taux de disponibilité en matériel informatique (ordinateur) était à 100% dont la majorité en mauvais état (67%).

**Figure 2:**
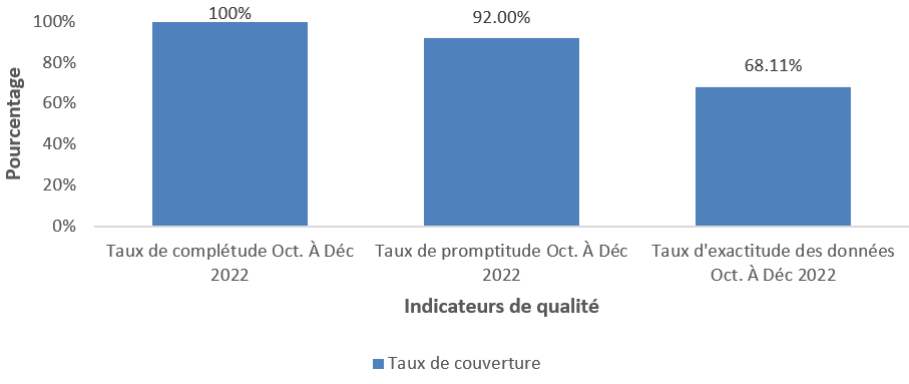
dimensions de la qualité des données

**Qualité des données du SISR:** notre étude a noté un taux de complétude à 100% aussi bien en milieu urbain que rural. Le taux de promptitude était à 100% pour le milieu rural et 83% pour le milieu urbain pour une promptitude globale moyenne de 92% (Tableau 1). L'exactitude des données au niveau des formations sanitaires a été calculée en comparant les données rapportées par les formations sanitaires visitées et celles recomptées dans les différents outils de collecte des données des indicateurs de revue (registres, fiches, rapports mensuels des activités). Il ressort que tous les indicateurs présentent une moyenne d'exactitude des données qui varie entre 56% à 98%. Nous observons que l'exactitude est meilleure au milieu urbain pour les deux premiers indicateurs (MAS sans complications (100%) et CPN4 (59%) et meilleur au milieu rural pour les derniers indicateurs (paludisme simple chez les moins de 5 ans (99%) et penta 3 chez les moins de 1 an (95%). Parmi les trois dimensions de la qualité des données évaluées dans cette étude, une complétude de 100%, une promptitude de 92% et une exactitude de 68,11% ont été observés parmi les formation sanitaires étudiées (Figure 2).

**Tableau 1:** répartition des taux de complétude et de promptitude des données selon les formations sanitaires

Formations sanitaires	Rapports attendus	Rapports complets	Pourcentage (%)	Envoyés dans le délai	Pourcentage (%)
Aglal	12	12	100	12	100
Bellefarandi	12	12	100	12	100
Ber	12	12	100	12	100
Hondoubomo Koïna	12	12	100	12	100
Kabara	12	12	100	12	100
Sankoré	12	12	100	6	50
Total	72	72	100	66	92

**Les contraintes influençant le SISR:** seulement 51,1% des agents de santé sont impliqués dans la collecte avec un ratio personnel impliqué dans la collecte par formation sanitaire de 4,5 et seul les DTC (22,22%) font la saisie dans le Dhis2 avec un ratio personnel impliqué dans la saisie Dhis2 par formation sanitaire de 1.

Près d'un tiers (30% et 33%) des répondants ont déclaré avoir bénéficié de formation respectivement sur le SISR et sur l'utilisation de la plateforme Dhis2 au cours des 6 derniers mois. Près de 63% ont déclaré avoir reçu une supervision dont 47% à deux reprises le trimestre dernier, mais seulement 26% ont reçu une retro-information.

Près des deux tiers (67%) des répondants ont déclaré qu'il n'y a pas d'accès à internet en permanence dans les formations sanitaires. Les véhicules de transport en commun hebdomadaire (33%), les motos (33%) et le réseau social WhatsApp (28%) sont les moyens de transmissions des données les plus utilisés pour le niveau hiérarchique. Près de 90% font la saisie des données au sein des Organisation Non Gouvernementales (ONG) partenaires disposant de réseau satellitaire (VSAT).

Parmi les répondants, 37% ne se sentent pas en sécurité dans les formations sanitaires, 26% ont déclaré qu'il y a eu des braquages/enlèvements de leurs biens dans l'aire de santé le trimestre dernier pour une moyenne 5±4 par acte pendant le trimestre écoulé. La peur et le souci pour sa vie sont les sentiments les plus présents chez les répondants avec respectivement 62% et 15% (Tableau 2).

**Tableau 2:** récapitulatif des contraintes majeures et des stratégies de résilience adoptées par les acteurs

Contraintes majeures	Stratégies de résiliences
**Contraintes liées au personnel de santé**	
51,11% des agents de santé sont impliqués dans la collecte	Atelier mensuel de saisie de saisie des données au sein des ONG partenaires disposant des réseaux satellitaires
22,22% sont impliqués dans la saisie des données sur la plateforme DHIS2.	
**Contraintes liées à la formation, la supervision et la retro-information**	
29,63% et 33,33% des répondants ont déclaré avoir bénéficié de formation sur le SISR et sur l'utilisation de la plateforme DHIS2 au cours des 6 derniers mois;	33,33% des formations sanitaires ont formé un suppléant à la saisie des données dans le DHIS2
63% ont déclaré avoir reçu une supervision, 47,06% ont bénéficié d'au moins deux fois sur les 3 derniers mois et 25,93% ont reçu une retro-information.	Aucune stratégie n'a été retrouvé
**Contraintes liées à la connexion**	
33,33% des formations sanitaires ont accès à l'internet en permanence.	Les moyens de transmissions des données au niveau hiérarchique: véhicules de transport en commun des foires hebdomadaire (33,33%), motos (33,33%) et réseau social WhatsApp (27,78%);
	90% font la saisie mensuelle des données au sein des ONG partenaires disposant de réseau satellitaire (VSAT).
**Contraintes liées à la sécurité**	
37,04% ne se sentent pas en sécurité dans les formations sanitaires	50% quittent les formations en dehors des horaires de travail
25,93% ont déclaré qu'il y a eu des attaques/ braquages/enlèvement des biens dans votre aire (zone) de santé le trimestre dernier	50% évitent les routes les jours de foire hebdomadaires (rural)
61,76% des répondants ont peur et 14,71% ont le souci pour leur vie	100% croient à la protection de Dieu

## Discussion

Cette étude visait à étudier la gestion du SISR à travers son fonctionnement, la qualité des données et les contraintes majeures, ainsi que les stratégies de résilience des acteurs du SIS pour maintenir la performance du SIS en situation de crise sécuritaire dans le district sanitaire de Tombouctou parce que malgré les appuis des ONG partenaires du district et de l'État, nous observons des difficultés majeures rencontrées par ces agents de santé. C'est l'une des premières enquêtes qui s'est intéressée sur les aspects de gestion des données dans cette partie du pays (septentrion malien).

**Processus de fonctionnement du SIS:** notre étude a obtenu un taux de disponibilité des directives nationales à 83%, des supports (registres et rapports) à 100%, ainsi qu'un taux de rupture des supports à 17% sur les six derniers mois. Nos résultats sont comparables à ceux du niveau national en 2018 qui a obtenu un taux disponibilité qui oscillait entre 63 et 99% pour la disponibilité des supports, et entre 3 et 19% pour la rupture en supports de gestion [22]. Erick *et al*. en 2015 en RDC a obtenu des résultats similaires [20]. Ce taux de rupture pourrait s'expliquer par l'insuffisance des supports envoyés par niveau, le retard de la mise à disposition et d'acheminement des supports du niveau national au niveau local.

Concernant le processus proprement dit du SISR, les taux en matière de collecte des données (33,33%), d'analyse des données (33,33%) et de rétro-information (0%) sont à des niveaux très faibles (moins de 50%) pour des scores relativement meilleurs pour le traitement (50%), la transmission (83,33%), l'affichage des données (100%), la tenue (83,33%) et la disponibilité des comptes rendus des réunions (supérieur ou égal à 50%). Des résultats similaires aux nôtres ont été obtenu dans des enquêtes nationales comme celle de la Guinée en 2014 [35] et du Burundi en 2009 [36]. Quant aux matériels informatiques (ordinateur), toutes les formations sanitaires en disposaient (100%) dont deux tiers (66,67%) en mauvais état. Nos chiffres sont supérieurs à ceux de Shama *et al*. en 2021 dans la région de Harari en Éthiopie, qui a obtenu 30,6% pour la disponibilité des matériels informatiques [37]. Il faut noter que la plupart de ces ordinateurs datent de 2016.

**Qualité des données du SISR:** notre étude a obtenu un taux de complétude à 100% aussi bien en milieu urbain que rural. Ce résultat est supérieur à celui de plusieurs études: l'enquête nationale en 2018 (84%) [22], Shama *et al*. en Éthiopie en 2021 (60%) [37] et Erick *et al*. en 2015 en RDC (76%) [20]. Quant au taux de promptitude, il était de 100% en milieu rural et 85% pour le milieu urbain, et la promptitude globale de l'étude était de 92%. Ce taux comparable à celui obtenu par Shama *et al*. en 2021 en Éthiopie, soit 93,7% [37]. Des taux plus faibles au nôtre ont été obtenus par d'auteurs: l'évaluation du niveau nationale en 2018 (51%) [22], Kebede *et al*. en 2020 en Éthiopie (72,2%) [38] et au Burkina Faso en 2020 (82%) [19]. Notre taux est une bonne performance par rapport aux recommandations du Ministère de la Santé qui requiert qu'au moins 90% des rapports doivent être saisie dans le délai. Ces résultats satisfaisants pourraient s'expliquer par les efforts fournis par les ONG partenaires qui financent des rencontres mensuelles de saisie des données dans le Dhis2 la première semaine du mois suivant de façon régulière.

Notre étude a mis en évidence que 68,11% des formations sanitaires ont un taux d'exactitude répondant aux normes. Notre résultat est supérieur à celui du niveau national en 2018 (45%) pour les formations sanitaires [22] et de celui obtenu par Shama *et al*. en Éthiopie en 2021 (58,10%) [37]. L'évaluation du SISR par les outils PRISM au Burundi en 2009 a eu un taux d'exactitude supérieur au nôtre (72%) [36]. L'analyse des résultats a révélé une grande disparité entre les structures et les indicateurs. Les sous-déclarations ou sur-déclarations de données seraient dues à deux facteurs principaux: l'absence de documents et des données manquantes dans les documents disponibles, ainsi qu'à des erreurs d'enregistrement et de compilation des données par les prestataires en surcharge de travail, insuffisant en nombre, faiblement outillé, une insuffisance de formation ciblées sur le SISR et parfois des faux rapports pour augmenter les résultats. Ils pourraient aussi s'expliquer par le fait que les agents de santé sont plus concentrés sur la gestion des patients que sur l'enregistrement des données en raison de la charge de travail et le manque d'engagement envers les données. D'autres études ont également révélé des incohérences des données similaires dans la gestion des données du SISR, notamment au Mali en 2018 [22], au Burkina-Faso en 2020 [19] et en Guinée en 2014 [35].

**Les contraintes influençant le SISR:** dans notre étude, seulement 51,11% du personnel sont impliqués dans la collecte avec un ratio personnel impliqué dans la collecte par formation sanitaire de 4,5 et seuls les responsables de formations sanitaires (22,22%) font la saisie dans le Dhis2 avec un ratio personnel impliqué dans la saisie Dhis2 par formation sanitaire de 1. Nos résultats concordent avec ceux de la dernière évaluation du niveau national en 2018, soit un ratio de 2,39 et 3,25 respectivement pour le personnel impliqué dans la saisie des données et dans la collecte des données [22]. Nos résultats pourraient s'expliquer par l'insuffisance en ressources humaines qualifiées, le manque de restitution de l'équipe du centre au retour de formations, ce qui entraine un désintéressement des autres agents sur les activités SISR et une surcharge de travail de ceux déjà impliqués dans la collecte des données du SIS.

Notre étude a démontré que près d'un tiers (29,63% et 33,33%) des répondants ont déclaré avoir bénéficié de formation respectivement sur le SISR et sur l'utilisation de la plateforme Dhis2 au cours des 6 derniers mois. Ces chiffres sont supérieurs à ceux du niveau de shama *et al*. en Éthiopie (14,9%) [37]. La formation peut fournir aux participants les connaissances et les compétences nécessaires pour utiliser efficacement les outils du SIS, tels que les registres, les formats de rapport et le logiciel Dhis2. Près de 63% ont déclaré avoir bénéficié de supervision dont 47,06% ont bénéficié d'au moins deux supervisions le trimestre dernier. Ces chiffres sont inférieurs à ceux de shama *et al*. en 2021 (77,5%) [37] et du niveau national en 2018 [22]. Seulement 25,93% ont reçu une retro-information après supervision, ce qui est comparable à celui obtenu par Erick *et al*. (33%) [20], mais inférieur à celui de shama *et al*. (61,7%) [37]. La justification possible est que dans la plupart des cas pratiques, la supervision est souvent vécue par le personnel de santé comme une source de stress et d'anxiété, plutôt qu'une opportunité de s'améliorer. La supervision formative ciblée permet aux formations sanitaires d'identifier et de combler leurs lacunes en matière de gestion des données.

Notre étude a montré que 2 formations sanitaires sur 3 (66,67%) n'avaient pas accès à internet en permanence et même quand il est présent, c'est de mauvaise qualité. Ce résultat est comparable à celui du niveau national en 2018 (69%) [22]. Shama *et al*. dans une étude réalisée en Éthiopie sur un échantillon 314 départements de santé de la région de Harari en 2021, l'accès à internet était de 31% [37]. Ces difficultés d'accès à internet dans notre contexte sont dues à l'instabilité du réseau provoquée par des bandits armés qui s'adonnent à des sabotages des antennes relais et enlèvement des panneaux solaires et des groupes électrogènes qui les alimentent, perturbant ainsi la qualité du réseau dans presque toute la région de Tombouctou. Malgré ces difficultés d'accès à internet, les agents de santé arrivaient à envoyer au niveau hiérarchique et saisir les données régulièrement, le plus souvent à temps. Ainsi 33,33% envoyaient les données et les copies dures des rapports mensuels par le biais des véhicules de transport en commun des foires hebdomadaires et des motos faisant la liaison entre les villages et la ville. Une autre stratégie est l'envoi des données par le réseau social WhatsApp (27,78%). Quant-à la saisie des données dans le Dhis2, 90% des agents de santé impliqués dans la saisie des données se déplaçaient en ville au sein des ONG partenaires disposant de réseau satellitaire pour la saisie des données dans le Dhis2.

Dans notre étude, environ 37,04% déclaraient ne pas se sentir en sécurité dans les formations sanitaires, 25,93% avaient déclaré des cas braquages ou enlèvements des biens dans l'aire de santé le trimestre dernier. La peur et le souci pour sa vie sont les sentiments les plus présents avec respectivement 61,76% et 14,71%. Ces sentiments sont les plus retrouvés dans beaucoup d'étude sur les travailleurs de la santé en zone de conflits en plus de stress traumatique, l'anxiété, la dépression, la colère, etc. [39]. Ce sont les cas d'une étude réalisée en 2014 par Abu-El-Noor *et al*. sur les troubles de syndrome de stress post-traumatique (SSPT) chez les prestataires de soins de santé à la suite des attaques israéliennes contre la bande de Gaza, dont 54,85% des infirmières et 47,38% des médecins avaient de stress suite à la peur constante [40]. Il en est de même pour une étude réalisée par Sharon *et al*. suite aux attentats du 11 septembre 2001 aux États-Unis où 44% de la population adulte avait connu un stress, une peur et une insécurité importante, ainsi que des taux plus élevés de SSPT [41]. Il faut noter que les formations sanitaires sont de plus en plus des cibles d'attaques, enlèvements, vols, extorsion, etc., rendant ainsi les conditions de travail très difficile.

**Limites et biais de l'étude:** la taille de notre étude pourrait constituer une limite bien que nous ayons pris en compte aussi bien le milieu rural qu'urbain. En effet, la plupart des études dans le domaine sont faites sur une étendue de pays, de région ou de province avec le plus souvent des échantillons de plus de 100 répondants. Nos résultats ne sont pas généralisables à l'échelle nationale, mais ils sont pertinents pour la région de Tombouctou et des contextes similaires. Le caractère déclaratif des impressions et des ressentiments par rapport au contexte sécuritaire peut constituer un biais d'information.

## Conclusion

Cette étude nous a permis de comprendre le niveau de fonctionnement du SIS en situation de crise sécuritaire, d'analyser la qualité des données et d'identifier les contraintes majeures, ainsi que les stratégies de résilience des acteurs du SIS afin de fournir des informations vérifiables sur le phénomène aux membres de la communauté scientifique. Ces résultats ont démontré que plusieurs aspects du processus de fonctionnement du SISR ont des difficultés notoires exacerbées par la crise sécuritaire que vivent les populations du septentrion malien. Ils présentent un faible niveau de processus, des matériels informatiques en mauvais état, une mauvaise couverture réseau et une insuffisance en personnel qualifié pour la gestion du SIS. Les problèmes liés à l'insécurité ont été également mis en exergue. Comprendre le fonctionnement du SIS et les contraintes que vivent les agents de santé en situation de crise, c'est une occasion de proposer des mesures qui permettront d'améliorer sa gestion. La gestion des données du SISR est un phénomène complexe en situation de crise avec son lot de conséquences sur la performance du SIS ainsi que la santé mentale des agents de santé sur le terrain. Elle devrait dans l'avenir faire l'objet d'une étude plus en approfondie prenant en compte ses facteurs à travers des études mixtes sur une échelle plus grande au Mali.

### Etat des connaissances sur le sujet

- *Le phénomène de la gestion des données du SISR est étudié dans le monde et les cadres de métrologie sanitaire et de PRISM a été défini par l'OMS et measure evaluation;*
- *Le SIS est l'un des six piliers essentiels du système de santé pour atteindre l'objectif de la santé pour tous établi par l'OMS;*
- *Il n'est pas assez étudié dans les pays en développement, avec très peu d'étude en Afrique*.

### Contribution de notre étude à la connaissance

- *Notre étude apporte pour la première fois le niveau de fonctionnement des processus SIS en situation de crise sécuritaire dans le district de Tombouctou;*
- *Elle décrit les contraintes majeurs et les stratégies de résilience des acteurs du SIS en situation de crise sécuritaire*.

## Remerciements

À la Direction Régionale de la Santé et au District Sanitaire de Tombouctou pour la facilitation de l'enquête sur terrain. À l'ONG John Snow Inc., pour avoir permis de mener l'étude durant la période de stage.

## References

[1] Organisation mondiale de la Santé (2021). Système de santé contribuant à la sécurité sanitaire.

[2] World Health Organization (2007). Everybody´s business - strengthening health systems to improve health outcomes: WHO´s framework for action. World Health Organization.

[3] Willis CD, Barbara LR, Carol PH, Allan B (2013). Networks to Strengthen Health Systems for Chronic Disease Prevention. Am J Public Health.

[4] Health Metrics Network, Organization World Health (2008). Framework and standards for country health information systems. World Health Organization.

[5] Glèlè Ahanhanzo Y, Ouendo E-M, Kpozèhouen A, Levêque A, Makoutodé M, Dramaix-Wilmet M (2015). Data quality assessment in the routine health information system: an application of the Lot Quality Assurance Sampling in Benin. Health Policy Plan.

[6] Checchi F, Warsame A, Treacy-Wong V, Polonsky J, van Ommeren M, Prudhon C (2017). Public health information in crisis-affected populations: a review of methods and their use for advocacy and action. Lancet.

[7] Olu O (2017). Resilient Health System As Conceptual Framework for Strengthening Public Health Disaster Risk Management: An African Viewpoint. Front Public Health.

[8] Cecchi-Teneerini ML, Laffon P (2002). Evaluation du système d´information des professionnels de la santé.

[9] Dehnavieh R, Haghdoost A, Khosravi A, Hoseinabadi F, Poursheikhali A (2019). The District Health Information System (DHIS2): A literature review and meta-synthesis of its strengths and operational challenges based on the experiences of 11 countries. Health Inf Manag.

[10] Kraj ČA T (2010). Composants pour le système d´information sanitaire DHIS2. Masarykova univerzita, Fakulta informatiky.

[11] Garrib A, Stoops N, McKenzie A, Dlamini L (2008). An evaluation of the District Health Information System in rural South Africa. S Afr Med J.

[12] Organisation Mondiale de la Santé Systèmes d´information sanitaire à l´appui des objectifs du Millénaire pour le développement 2006 mai5 Report No.: EB118/16.

[13] Aqil A, Lippeveld T, Hozumi D (2009). PRISM framework: a paradigm shift for designing, strengthening and evaluating routine health information systems. Health Policy Plan.

[14] Konan DJP, Aka J, Yao KJ, Kouassi-Gohou V, Yao KE, Faye-Kette H (2013). Le point sur une maladie tropicale négligée à travers le système d´information sanitaire de routine en Côte d´Ivoire: le pian de 2001 à 2011. Médecine et Santé Tropicales.

[15] Koumamba A (2021). Modèle de système d´information pour le pilotage. les statistiques et la veille sanitaire au Gabon [PhD Thesis]. Bordeaux.

[16] Ministère de la Santé et de l´hygiène publique Ivoirien (2009). Rapport d´évaluation du système national d´information sanitaire ivoirien par l´outil du réseau de métrologie sanitaire.

[17] Moussa L, N’Gbichi JM, Lippeveld T, Ye Y Rapport d'évaluation de la performance du Système d'Information Sanitaire de Routine (SISR) et de la Surveillance Intégrée de la Maladie et la Riposte (SIMR).

[18] Muhemedi S, Kabangu Y, Mpeli F, Salumu S, Kabeya P, Okitolonda E (2017). Evolution du système national d´information sanitaire de la république démocratique du Congo entre 2009 et 2015. Pan Afr Med J.

[19] Kebe MR, Ouangaré A, Tohouri RR, Kouassi C, Barry MA, Chauffour J Évaluation de la Performance du Système d´Information Sanitaire de Routine (PRISM) au Burkina Faso.

[20] Erick TM Analyse de la qualité des données du Système d´Information Sanitaire de routine: Défis et Perspectives pour un Système performant dans la Province du Maniema en République Démocratique du CONGO.

[21] OMS Mali (2013). L´impact de la Crise Humanitaire sur les Structures de Santé au Mali.

[22] Traoré A, Ba Kouyaté M, Maiga A, Ouatara A, Dembélé I, Doumbia A Évaluation de la performance du système local d´information sanitaire au Mali 2018.

[23] Ministère de la santé et de l'hygiène publique (2014). Évaluation du système local d'information sanitaire avec les outils PRISM. Mali.

[24] MEASURE Evaluation Evaluation des Niveaux CSREF. CSCOM et Communautaire du SLIS du Mali a l´Aide de l´Outil PRISM.

[25] MEASURE Evaluation L'effet boule de neige de la compétition sur la qualité des données sanitaires au Mali.

[26] MEASURE Evaluation L'expérience du Mali dans le déploiement du DHIS2.

[27] MEASURE Evaluation Routine Data Quality Assessment (RDQA) Curriculum Materials.

[28] Statistics Canada Data quality toolkit.

[29] Dumont A, Gueye M, Sow A, Diop I, Konate MK, Dambé P (2012). Utilisation des données recueillies en routine pour évaluer l´activité des maternités au Mali et au Sénégal (essai QUARITE). Rev Épidémiol Santé Publique.

[30] Hardee K Outil d´évaluation systématique de la qualité des données-- Manuel de Mise en Œuvre.

[31] MEASURE Evaluation (2022). Outils d'évaluation et d'amélioration de la qualité des données (RDQA et DQA). Consulté le 30 décembre.

[32] Statistique Canada Politique visant à informer les utilisateurs sur la qualité des données et la méthodologie.

[33] World Health Organization (WHO) Data quality review: module 1: framework and metrics.

[34] World Health Organization (2015). Data quality review (DQR): A toolkit for facility data quality assessment, Version 1.0.

[35] Direction Nationale du Bureau Stratégie et développement (République du Guinée) Évaluation du système d'information sanitaire de routine avec l'approche et les outils PRISM juin 2014.

[36] Direction Générale de la Planification, Direction du Système National d'Information Sanitaire (DSNIS) Rapport d'Evaluation du Système d'Information Sanitaire de Routine par l'approche et les outils PRISM.

[37] Shama AT, Roba HS, Abaerei AA, Gebremeskel TG, Baraki N (2021). Assessment of quality of routine health information system data and associated factors among departments in public health facilities of Harari region, Ethiopia. BMC Med Inform Decis Mak.

[38] Kebede M, Adeba E, Chego M (2020). Evaluation of quality and use of health management information system in primary health care units of east Wollega zone, Oromia regional state, Ethiopia. BMC Med Inform Decis Mak.

[39] Moreno-Chaparro J, Piñeros-Ortiz S, Rodríguez-Ramírez L, Urrego-Mendoza Z, Garzón-Orjuela N, Eslava-Schmalbach J (2022). Mental health consequences of armed conflicts in adults: an overview. Actas Esp Psiquiatr.

[40] Abu-El-Noor NI, Aljeesh YI, Radwan AS, Abu-El-Noor MK, Qddura IAI, Khadoura KJ (2016). Post-Traumatic Stress Disorder Among Health Care Providers Following the Israeli Attacks Against Gaza Strip in 2014: A Call for Immediate Policy Actions. Arch Psychiatr Nurs.

[41] Perlman SE, Friedman S, Galea S, Nair HP, Erős-Sarnyai M, Stellman SD (2011). Short-term and medium-term health effects of 9/11. Lancet.

